# Self-monitoring of Blood Glucose in Non-Insulin Treated Type 2 Diabetes (The SMBG Study): study protocol for a randomised controlled trial

**DOI:** 10.1186/s12902-017-0154-x

**Published:** 2017-01-26

**Authors:** Sharon Parsons, Stephen Luzio, Stephen Bain, John Harvey, Jillian McKenna, Atir Khan, Sam Rice, Alan Watkins, David R. Owens

**Affiliations:** 1 Diabetes Research Unit, Swansea University, Institute of Life Sciences, Singleton Park, Swansea, SA2 8PP UK; 2 Diabetes Centre, Wrexham Maelor Hospital, Betsi Cadwaladr University Health Board, Wrexham, UK; 3 Diabetes Centre, Glangwili Hosptial, Hywel Dda University Health Board, Carmarthen, UK; 4 Diabetes Centre, Prince Philip Hospital, Hywel Dda University Health Board, Llanelli, UK; 5 Swansea Trials Unit, Swansea University, Swansea, UK

**Keywords:** Structured SMBG, Type 2 diabetes, Insulin naïve, Randomised controlled trial

## Abstract

**Background:**

The benefit of Self-monitoring of Blood Glucose (SMBG) in people with non-insulin treated type 2 diabetes remains unclear with inconsistent evidence from randomised controlled trials fuelling the continued debate. Lack of a consistent finding has been attributed to variations in study population and design, including the SMBG intervention. There is a growing consensus that structured SMBG, whereby the person with diabetes and health care provider are educated to detect patterns of glycaemic abnormality and take appropriate action according to the blood glucose profiles, can prove beneficial in terms of lowering HbA1c and improving overall well-being. Despite this, many national health agencies continue to issue guidelines restricting the use of SMBG in non-insulin treated type 2 diabetes.

**Methods:**

The SMBG Study is a 12 month, multi-centre, randomised controlled trial in people with type 2 diabetes not on insulin therapy who have poor glycaemic control (HbA1c ≥58 mmol/mol / 7.5%). The participants will be randomised into three comparative groups: Group 1 will act as a control group and receive their usual diabetes care; Group 2 will undertake structured SMBG with clinical review every 3 months; Group 3 will undertake structured SMBG with additional monthly telecare support from a trained study nurse. A total of 450 participants will be recruited from 16 primary and secondary care sites across Wales and England. The primary outcome measure will be HbA1c at 12 months with secondary measures to include weight, BMI, total cholesterol and HbA1c levels at 3, 6, 9 and 12 months. Participant well-being and attitude towards SMBG will be monitored throughout the course of the study. Recruitment began in December 2012 with the last participant visit due in September 2016.

**Discussion:**

This study will attempt to answer the question of whether structured SMBG provides any benefits to people with poorly controlled type 2 diabetes who are not being treated with insulin. The data will also clarify whether the telecare support provides additional value. The overall acceptability of SMBG as a tool for self-management will be assessed.

**Trial registration:**

UKCRN 12038 (Registered March 2012).

ISRCTN21390608 (Retrospectively registered 15^th^ May 2014).

## Background

Self-monitoring of blood glucose (SMBG) is recommended as a core element of self-management of diabetes when used appropriately following suitable training [1, 2]. In persons requiring insulin therapy, the information gained from SMBG can be used to adjust lifestyle (nutrition and physical activity) and insulin doses to optimise glycaemic control. However, the benefit of SMBG in insulin naïve type 2 diabetes has not been a consistent finding in the limited number of randomised control trials (RCTs) published to date [3–12]. Consequently, NICE have recently issued guidelines [13] similar to the US, Canada and Australia [14], recommending limiting the use of SMBG in people with type 2 diabetes. However, current recommendations make allowances where there is evidence of hypoglycaemic episodes, the person is on oral medication that may increase their risk of hypoglycaemia while driving or operating machinery, during pregnancy, or when planning to become pregnant. If adults with type 2 diabetes are self-monitoring their blood glucose levels, NICE now recommends a structured assessment should be carried out at least annually.

The studies conducted on SMBG have varied in terms of their methodology, populations and intervention (format of SMBG). However, ‘structured SMBG’, involving regular ‘paired blood glucose testing’ (pre and post meal) to identify patterns of glycaemic control along with education to interpret the results and action taken to correct any abnormalities, has consistently demonstrated clear benefits with improved HbA1c and well-being [9, 14]. This approach is now generally recommended as the optimum method for blood glucose self- monitoring [1]. Clinical practice suggests that many with type 2 diabetes perform SMBG but do not act on the results thus underutilising its potential benefit in terms of necessary adjustment of lifestyle and/or dose of oral hypoglycaemic agents. Often, people with type 2 diabetes have not had the necessary education or training to adjust their lifestyle or oral medication even if they are aware that their blood glucose results are abnormal [15].

In 2014/15, NHS expenditure on blood glucose monitoring agents and devices in England was £175.2 million. This represents an increase of 23% since 2005/06 accounting for just over 20% of the total cost of diabetes treatments in England that year [16]. Despite the continued year on year rise in expenditure, HbA1c levels in people with type 2 diabetes have remained static with only approximately 66% reaching the NICE recommended target of 58 mmol/mol (≤7.5%) [17]. As a consequence the debate continues regarding the value of SMBG in people with type 2 diabetes who are not on insulin therapy [18–22].

## Methods and study design

### Aim

To demonstrate that a proactive, nurse-led service, using structured SMBG, can enable poorly controlled (HbA1c ≥58 mmol/mol / 7.5%) people with type 2 diabetes to better manage their diabetes. This is a randomised clinical trial comparing the use of structured SMBG when used alone, or with additional telecare (additional telephone support by trained nurses who will have the participants’ SMBG results available via an electronic upload system) versus no SMBG in people with type 2 diabetes not on insulin therapy which will serve as the control group. Throughout the trial, the well-being and satisfaction of participants will also be evaluated.

This study aims to determine if HbA1c is significantly improved at 12 months in participants who receive SMBG compared to the control group and also to determine which of the two SMBG regimens has the greater effect on reducing HbA1c.

### Study design

**Fig. 1:**
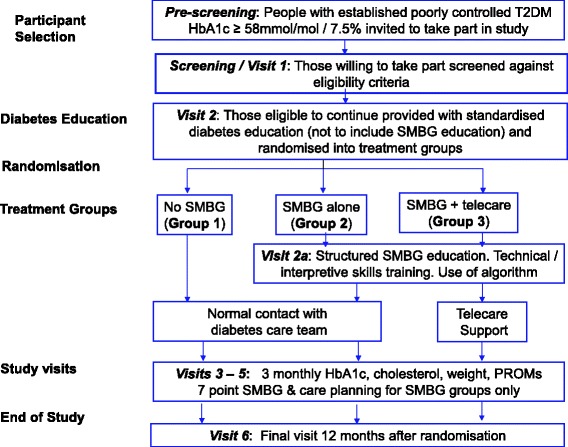
Study Design Flow Chart

The study is an open, multi-centre, randomized controlled trial (RCT). Participants are involved for 12 months following randomisation to (1) a control group with no SMBG monitoring, (2) structured SMBG alone and (3) structured SMBG with telecare, as illustrated in the Study Design Flow Chart (Fig. 1).

### Setting and site selection

The study is being conducted at primary and secondary care sites across Wales and England. Larger primary care (GP) practices in Wales, defined as those with a practice population greater than 9000, with over 500 people on their Diabetes Disease Register of whom 20% or more had sub-optimal blood glucose control according to Quality and Outcomes Framework (QoF) data, were initially approached to take part in the study as recruiting sites. Smaller, research active practices were also invited to participate. Diabetologists working in secondary care across Wales were invited to be involved by working in partnership with GP practices i.e. Patient Identification Centres. In addition, two sites in England, one in primary care and the other in secondary care, expressed an interest in taking part and were accepted, making a total of 16 centres (nine primary care sites and seven secondary care sites).

### Participants

Adults with established type 2 diabetes (>1 year duration) not receiving insulin therapy will be invited to take part in the study. Those recruited will meet the following inclusion/exclusion criteria:

- Inclusion criteria
  - Type 2 diabetes mellitus patients with a duration of diabetes >1 year;
  - Age ≥18<80 years;
  - HbA1c ≥58 to ≤119 mmol/mol (≥7.5% to ≤13%);
  - Willing and able to provide informed consent;
  - Access to a telephone;
  - Able to conduct blood glucose measurements.
- Exclusion criteria
  - Diabetes other than type 2 diabetes or type 2 diabetes treated with insulin;
  - Pregnancy;
  - Gestational diabetes mellitus;
  - Severe chronic hepatic disease;
  - Where using SMBG is part of their routine clinical care;
  - Participation in any investigational drug trial within 1 month prior to Visit 1;
  - Mental condition rendering the participant unable to understand the nature, scope and possible consequences of the study;
  - End-stage renal disease (existing or planned dialysis or transplantation) or creatinine >150 umol/L;
  - Blindness or severe loss of vision in both eyes.

Written informed consent will be given by the participant before any study activities take place. People with type 2 diabetes eligible to take part in the study will receive an invitation letter from their GP or hospital consultant, along with a Participant Information Leaflet and be given an oral explanation about the study from a research professional (usually a research nurse).

### Diabetes education

Following recruitment to the study all participants will be asked to complete the Audit of Diabetes Knowledge (ADKnowl) questionnaire to record their level of diabetes knowledge. Provision will be made to address any educational gaps through the use of standardised diabetes education materials. All participants will be given a copy of the Diabetes UK booklet on type 2 diabetes [23–25] that they will keep throughout the study duration to use for their personal reference. Those in the SMBG study arms (groups 2 and 3) will also be able to refer to this booklet at clinic visits when care planning with their study nurse.

### Randomisation

Following acceptance into the study, participants will be randomised into one of three treatment groups. The randomisation procedure uses study site and previous experience with SMBG (Yes/No) as stratifying factors, and aims to allocate approximately equal numbers of participants in the three groups overall, by site and by previous experience. Randomisation will be performed remotely by the Swansea Trials Unit (formerly West Wales Organisation for Rigorous Trials in Health and social care (WWORTH)). Randomisation will be via email using a central database.

### Study visits

The study will involve six visits for those randomised to the control group and an additional seventh visit for those randomised to one of the SMBG groups to deliver the SMBG training.

#### Visit 1 (Consent and screening visit)

At this visit and prior to any participant related activity, written informed consent is provided by the participant. Demographic data (such as sex, age, employment status) will be recorded and participants will be screened against the study inclusion/exclusion criteria. A blood sample will be taken to ascertain whether the HbA1c level is within the inclusion criteria. Following the visit all participants will be contacted to notify them of the outcome of the screening visit and those who meet the inclusion/exclusion criteria will be invited back for visit 2.

#### Visit 2 (Baseline and randomisation visit)

At visit two participants will be randomised into one of the three treatment groups. Participants will also be asked to complete the ADKnowl questionnaire [26] to record their current level of diabetes education. Standardised diabetes information (DUK Booklet [23–25]) will be given to all participants to take away with them. Baseline data will include collection of clinical data (e.g. height, weight, waist circumference), details of current treatment, blood sample for measurement of HbA1c and total cholesterol and participant reported outcomes (ADDQoL, EQ-5D & PHQ-9 questionnaires) [27–29]. For the SMBG groups there will also be a questionnaire assessing their attitude to SMBG.

Participants in all groups will be provided with a participant diary to record any significant events, change of medication and contact with any health care professionals.

#### Visit 2a (SMBG groups only)

Participants randomised to the SMBG groups will attend an individual training session with their study nurse on blood glucose monitoring teaching them how to monitor their blood glucose levels using the Accu-Chek Aviva meter. Participants will also have the option of using the Accu-Chek 360° Diabetes Management System and Accu-Chek 360° View Paper Tool (Roche Diagnostics GmbH, Mannheim, Germany). They will be taught blood glucose pattern recognition and how to use the algorithm supplied to self-adjust their lifestyle and/or treatment.

#### Visits 3–6 (3, 6, 9 and 12 months after visit 2)

During these visits the study nurse will carry out a review of the participant similar to Visit 2. Procedures will include collection of clinical data, details of current treatment, blood sample for measurement of HbA1c and total cholesterol, patient reported outcomes (EQ-5D & PHQ-9) and review of participant diaries. Participants will be asked to complete the ADDQoL questionnaire at visits 4 and 6 (6 and 12 month follow up visits) and the ADKnowl questionnaire at visit 6 (12 month follow up visit). For those in the SMBG groups, the study nurse will review the blood glucose readings and discuss and agree a care plan for the next 3 months at each visit. Participant diaries will be collected at the final visit.

The results from the study venous blood samples taken will not be fed back to the participant, the study nurse or any member of the participant’s health care team in order to keep the primary outcome measure (HbA1c) blinded across all treatment groups.

### The intervention

As the study nurses will vary according to their knowledge and expertise, all will attend a standardised training programme delivered by the study team in addition to completing online training covering the safe use of non-insulin therapies in type 2 diabetes. The standardised training programme will cover the correct technique for self-monitoring blood glucose, use of the Accu-Chek 360° View Tool and Accu-Chek 360° Diabetes Management System, glycaemic pattern recognition and use of the study specific participant and clinical algorithms. Refresher training will be provided approximately every 4 months as part of the study update meetings.

Throughout the study, participants in the control group (Group 1), will receive routine care with the participant able to contact their diabetes team or GP as they would normally. Group 1 participants will not be provided with a blood glucose meter and training on SMBG will not be given. Glycaemic management will be by their usual health care provider as part of routine clinical care.

Participants in the SMBG alone (Group 2) and SMBG with telecare (Group 3) groups will be supplied with a blood glucose meter and instructed how to take blood glucose readings correctly. They will be taught blood glucose pattern recognition using the Accu-Chek 360° View Tool and will be offered the Accu-Chek 360° Diabetes Management System software to use at home if they wish. Participants in the SMBG groups will be able to understand their results and will have the ability to adjust their lifestyle and/or medication based on SMBG targets using an algorithm. Actions taken in response to the blood glucose monitoring will be recorded. At each study visit glycaemic management will be based on SMBG results alone and refresher training will be given on using the blood glucose meter correctly, understanding SMBG profiles and following the algorithm. Additionally the blood glucose meter will be calibrated.

In addition to the education and support given to the Group 2 participants (SMBG Alone), Group 3 participants (SMBG with Telecare) will be in regular contact with their study nurse. Between the 3 monthly study visits the participants will verbally report or upload their SMBG results to the database (if using the software) for the nurse to review on a monthly basis. Each month, the study nurse will contact the participant by phone and review the blood glucose readings, discussing any trends of glycaemic abnormalities. A care plan is then devised and agreed with the participant for the coming month.

Initiation and adjustment of therapy will be based on the consensus statement from the ADA/EASD [30]. The aim of treatment will be to obtain HbA1c 53 mmol/mol (<7%) and fasting glucose <6.0 mmol/L and 2 h post-prandial glucose <10 mmol/L. Initiation of insulin will be considered if HbA1c is 69 mmol/mol (>8.5%), however, once a participant starts on insulin they will no longer participate in the study.

Blood glucose measurements will be taken on two days of every week during the study by all the participants in both SMBG groups (groups 2 & 3) which will include a weekday and a day at the weekend. Blood glucose will be measured fasting and 2 h after breakfast, and pre and 2 h post the evening meal. In addition, in the week prior to clinic attendance (study visit) participants will perform a 7 point blood glucose profile (pre and 2 h following major meals, and bedtime) on three days. At the clinic visits the meters will be downloaded and calibrated.

### Efficacy measures

The primary efficacy measure will be HbA1c at 12 months.

Secondary efficacy measures will include HbA1c at 3, 6 and 9 months; Total cholesterol at 3, 6, 9 and 12 months; Weight; BMI; Waist circumference; Hypoglycaemia (symptomatic/confirmed/nocturnal/severe - requiring third party involvement); Hyperglycaemic events; Time to insulin treatment; medication use; Health-related utility (EQ-5D), disease-specific quality of life (ADDQoL), depression score (PHQ-9) and use of health care resources; Percentage of persons achieving target of HbA1c ≤ 53 mmol/mol (≤7%); Time to reach HbA1c target; Acceptability of SMBG (measured by SMBG8/SMBG14 questionnaire).

### Safety evaluations and data monitoring

The Data Monitoring Committee (DMC) will monitor the overall conduct of the trial, safeguarding the interests of the trial participants and assessing the safety and efficacy of the intervention. The HbA1c results reported weekly by the accredited central laboratory will be monitored by a sub-committee of the DMC to ensure any participant in the control group whose HbA1c level deteriorates by more than 15% over a 6 month period or exceeds 119 mmol/mol (13%) is flagged to their GP via their local study site. The actual result will not be reported to the local study team or any member of the participant’s health care team until the participant has completed the study.

### Sample analysis

All samples will be analysed in a central accredited diabetes laboratory, the Diabetes Research Unit Cymru laboratory based at Swansea University. HbA1c will be measured using both the Diabetes Control and Complications Trial (DCCT) aligned method and the International Federation of Clinical Chemistry and Laboratory Medicine (IFCC) standardised values [31].

### Statistical analysis plan

The study will be analysed on a comparative basis. All significance tests will be two tailed and carried out at the 5% level. All available data from withdrawn subjects will be included in the analysis up to the time of withdrawal where possible. All statistical hypothesis tests will be performed at a 5% significance level.

The primary objective of the trial will involve the analysis of HbA1c values at 12 months. These values will be checked for Normality, applying suitable transformations as necessary. The values will then be analysed using a general linear model with baseline values and a nested set of group identifiers included as explanatory covariates and factors. The efficacy of the intervention will be assessed by appropriate hypothesis tests on parameters for group identifiers, with suitable allowance for multiple comparisons.

Variables associated with secondary efficacy measures will be summarised and analysed using the approach outlined above for the primary measure, with linear models used for continuous outcomes and binary logistic regression models used for binary outcomes.

### Sample size

Initial sample size considerations were based on previous data in which the response within each subject group was normally distributed with standard deviation of 13 mmol/mol HbA1c (1.2%). If the true difference in the experimental and control means is 0.3%, (3 mmol/mol) (corresponding to an effect size of 0.25), we will need to study 378 experimental subjects and 189 control subjects to be able to reject the null hypothesis that the population means of the experimental and control groups are equal with probability (power) 0.8. The Type I error probability associated with this test of this null hypothesis is 0.05. In summary, allowing for up to approximately 33% drop-out (to include participants who convert to insulin), a total of 850 participants are required (*n* = 284 per treatment group).

Following the recruitment of the first participants, although no formal interim analysis was proposed or undertaken, initial routine checks on data quality following recruitment of the first wave of participants provided an opportunity to review some of the assumptions underpinning our original sample size calculations. It became obvious that if we remained with our initial inclusion criteria of HbA1c (≥64 mmol/mol/ ≥8% to ≤97 mmol/mol/ ≤11%) a large number of participants would be missed. We therefore modified the criteria to ≥58 mmol/mol (7.5%) to ≤119 mmol/mol (13%). The review also showed that, first, the drop-out rate was likely to be smaller than originally expected and, second, that our original estimate of an effect size of 0.25 seemed to be conservative, with evidence to support increasing this to 0.333.

With all other elements in sample size considerations held fixed, the combined effect of these changes means that a total sample size of between *n* = 398 and *n* = 424 would be sufficient to achieve the study aims. Our revised target is now 450 (*n* = 150 per treatment group), which is higher and enables us to be confident that the proposed sample size will prove to have sufficient statistical power for our planned analyses and reports.

## Discussion

The debate still continues regarding the effectiveness and value for money of SMBG in people with type 2 diabetes who are not receiving treatment with insulin. A number of diabetes disease management programmes have been developed with emerging technologies supporting patient-management processes. Internet based systems where patients upload blood glucose results which are reviewed by health care professionals (HCPs) have been shown to improve diabetes control over a short term [32]. In this randomised clinical trial, we intend to assess structured SMBG in this population by comparing no SMBG monitoring (control), SMBG alone, and SMBG with telecare. This paper summarises the current approved protocol in use at 16 participating centres across Wales and England.
